# Semi-implicit Non-conforming Finite-Element Schemes for Cardiac Electrophysiology: A Framework for Mesh-Coarsening Heart Simulations

**DOI:** 10.3389/fphys.2018.01513

**Published:** 2018-10-30

**Authors:** Javiera Jilberto, Daniel E. Hurtado

**Affiliations:** ^1^Department of Structural and Geotechnical Engineering, School of Engineering, Pontificia Universidad Católica de Chile, Santiago, Chile; ^2^Institute for Biological and Medical Engineering, Schools of Engineering, Medicine and Biological Sciences, Pontificia Universidad Católica de Chile, Santiago, Chile

**Keywords:** non-conforming finite elements, computational cardiology, cardiac electrophysiology, conduction velocity, nonlinear finite elements

## Abstract

The field of computational cardiology has steadily progressed toward reliable and accurate simulations of the heart, showing great potential in clinical applications such as the optimization of cardiac interventions and the study of pro-arrhythmic effects of drugs in humans, among others. However, the computational effort demanded by *in-silico* studies of the heart remains challenging, highlighting the need of novel numerical methods that can improve the efficiency of simulations while targeting an acceptable accuracy. In this work, we propose a semi-implicit non-conforming finite-element scheme (SINCFES) suitable for cardiac electrophysiology simulations. The accuracy and efficiency of the proposed scheme are assessed by means of numerical simulations of the electrical excitation and propagation in regular and biventricular geometries. We show that the SINCFES allows for coarse-mesh simulations that reduce the computation time when compared to fine-mesh models while delivering wavefront shapes and conduction velocities that are more accurate than those predicted by traditional finite-element formulations based on the same coarse mesh, thus improving the accuracy-efficiency trade-off of cardiac simulations.

## 1. Introduction

Computer simulations of the electrical activity of the heart have increasingly gained attention in the medical community, as they have steadily shown potential in the study of cardiac diseases and in the design of novel cardiac therapies. Current models of the human heart are able to represent the complex three-dimensional anatomical structure of the heart chambers, incorporating key functional features such as the Purkinje network and the cardiomyocyte orientation (Vadakkumpadan et al., 2009). Such advanced representation of the heart has enabled novel *in-silico* studies of undesired pro-arrhythmic effects of drugs in patients (Sahli Costabal et al., 2018), potentially reducing the number of subjects needed in a clinical trial by aiding the experiment design. Computational models of the heart have also shown promise in assisting the design of effective therapies for terminating atrial fibrillation (Trayanova et al., 2018). While these examples can only confirm the tremendous relevance of computational models in advancing the field of cardiology, they share the fundamental challenge of being highly demanding in terms of wall-clock time needed in computer simulations.

Mathematical models of the heart require the computer implementation of spatio-temporal discretization techniques in order to obtain a sequence of numerical representations of the physiological fields under study. Two fundamental aspects directly responsible for the computation time (CT) in a heart simulation are the ionic model used to account for subcellular electrochemical mechanisms, and the level of spatio-temporal discretization in terms of time-step size and mesh size (Sundnes et al., 2006). The choice of the mesh size typically faces a well-known trade-off problem of accuracy vs. efficiency, as decreasing the mesh size in a simulation results in more accurate numerical approximations, at the cost of increasing the number of degrees of freedom (DOFs), which drives the CT. Indeed, current simulations of the heart typically employ mesh sizes in the range of tens to hundreds of micrometers for domains with lengths in the order of centimeters, which ultimately translates into large systems of equations with several millions of DOFs that need to be solved at each time step. Such high dimensionality renders the solution of heart simulations extremely challenging for personal computers, and calls for improving their implementation in high-performance computing (HPC) platforms (Niederer et al., 2011a; Vazquez et al., 2011).

In the particular case of cardiac electrophysiology simulations, a common criterion to select the mesh size is the ability of the numerical simulation to recover an accurate conduction velocity (CV) and wavefront shape (Pathmanathan et al., 2010; Krishnamoorthi et al., 2013; Dupraz et al., 2015). It has been shown that both the wavefront shape and the CV suffer from a strong dependence on the spatial discretization, which for the case of finite-element (FE) discretization using linear basis functions results in a significant loss of accuracy for the case of mesh sizes >0.1mm (Pezzuto et al., 2016). In order to achieve larger mesh sizes, higher-order FE formulations have been proposed, which show that FE Lagrange basis functions of order 2 and 3 result in accurate CV for coarser meshes (Arthurs et al., 2012; Pezzuto et al., 2016). It should be noted, however, that higher-order FE schemes based on Lagrange basis functions necessarily increase the total number of DOFs in simulations when compared to linear-element formulations, as well as they require an additional computational effort for quadrature procedures, as higher-order basis functions demand the use of more quadrature-point evaluations (Cantwell et al., 2014). Recently, Hurtado and Rojas (2018) introduced a non-conforming finite-element scheme (NCFES) for the spatial discretization of the monodomain equation of cardiac electrophysiology that allows for the use of coarse meshes without significant loss of accuracy measured in terms of CV and wavefront shape. More specifically, hexahedral trilinear elements (Q1) were enhanced with non-conforming basis functions of degree 2 to create a non-conforming element (Q1NC) that is capable of representing a second-order polynomial within the element domain, a concept widely employed in the context of solid mechanics FE simulations (Wilson et al., 1973; Taylor et al., 1976). Further, they showed that the DOFs associated to the non-conforming basis functions can be solved at the element level, and therefore the number of global DOFs of the Q1NC scheme equals that of a standard Q1 FE scheme. As a result, Q1NC simulations delivered a CV and wavefront shape similar to that of second-order Lagrange formulations (Q2) at the computational cost in the order of a Q1 formulation.

During the development of the NCFES for cardiac electrophysiology, a fully-implicit (FI) backward-Euler time-stepping method was considered (Hurtado and Rojas, 2018). While FI schemes have important advantages in delivering a larger time-step stability region in cardiac simulations (Ying et al., 2008; Hurtado and Henao, 2014), they require the solution of a large system of non-linear equations at each time step that can be very costly in computational terms, and may not be well-suited to parallel-computing platforms when compared to other numerical schemes. To improve the computational efficiency, the semi-implicit integration method has been proposed in the literature for solving the semi-discrete equations resulting from standard FE discretizations, showing a relevant decrease in the CT of cardiac simulations, as well as being amenable to HPC platforms (Whiteley, 2006; Pathmanathan et al., 2010). Consequently, the scientific question that motivates this work is: Can we further improve the efficiency-accuracy trade-off in cardiac simulations by combining non-conforming FE spatial discretizations with semi-implicit time-integration schemes? To answer such question, in the following we develop the numerical framework and present an algorithm for the implementation of a semi-implicit non-conforming FE scheme to solve the monodomain electrophysiology equations, and investigate the numerical consequences and potential contributions to cardiac simulations.

## 2. Methods

### 2.1. Monodomain model of cardiac electrophysiology

Let Ω ∈ ℝ^3^ be the heart domain where electrical impulses travel during the time interval [0, *T*], and *V*_m_:Ω × [0, *T*] → ℝ be the transmembrane potential. A local statement of current balance yields the monodomain equation (Pullan et al., 2005)

(1) $$\begin{array}{l} A_{\text{m}}\left(C_{\text{m}}\frac{\partial V_{\text{m}}}{\partial t}+I_{\text{ion}}\left(V_{\text{m}},r\right)\right)-\text{div}\left(\sigma \nabla V_{\text{m}}\right)=0,\;\text{in}\Omega \times \left(0,T\right], \end{array}$$

where *A*_m_, *C*_m_ are the surface-to-volume ratio and membrane capacitance, respectively, σ is the conductivity tensor, *I*_ion_ is the ionic current depending on the transmembrane potential *V*_m_, and $r:\Omega \times \left(0,T\right]\to {\mathbb{R}}^{\text{m}}$ is a vector field of state variables that include gating variables and ion concentrations. For convenience, we consider the normalized transmembrane potential field

$$\begin{array}{l} \phi \left(x,t\right)=\frac{V_{\text{m}}\left(x,t\right)-V_{\text{r}}}{V_{\text{p}}-V_{\text{r}}}, \end{array}$$

where *V*_p_ and *V*_r_ are the peak and resting voltages, respectively. Based on this normalization, we obtain the non-dimensional monodomain equation,

(2) $$\begin{array}{l} \frac{\partial \phi }{\partial t}-\text{div}\left(D\nabla \phi \right)-f\left(\phi ,r\right)=0\;\text{in}\Omega \times \left(0,T\right], \end{array}$$

where $D=\frac{1}{A_{\text{m}}C_{\text{m}}}\sigma$ is the normalized conductivity tensor, and $f\left(\phi ,r\right)=-\frac{I_{\text{ion}}\left(V_{\text{m}}\left(\phi \right),r\right)}{C_{\text{m}}\left(V_{\text{p}}-V_{\text{r}}\right)}$ is the normalized ionic current. The time evolution of state variables is governed by kinetic equations of the form

(3) $$\begin{array}{l} \frac{\partial r}{\partial t}=g\left(\phi ,r\right)\;\text{in}\Omega \times \left(0,T\right]. \end{array}$$

The expressions for f(ϕ,r) and g(ϕ,r) will depend on the choice of ionic model representing the transmembrane ionic current in a single cell. Equations (2, 3) are complemented with Dirichlet and Neumann boundary conditions,

(4) $$\begin{array}{l} \phi =\bar{\phi },\;\text{on}\partial \Omega_{\phi }\times \left(0,T\right], \end{array}$$

(5) $$\begin{array}{l} q\cdot n=\bar{q},\;\text{on}\partial \Omega_{q}\times \left(0,T\right], \end{array}$$

respectively, as well as initial conditions

$$\begin{array}{l} \phi \left(x,0\right)=\phi_{0}\left(x\right),\;x\in \Omega ,\\ r\left(x,0\right)=r_{0}\left(x\right),\;x\in \Omega . \end{array}$$

To state the weak form of the cardiac electrophysiology problem, we consider trial spaces $S^{\phi },S^{r}$ and test spaces $V^{\phi },V^{r}$ defined as

(6) $$\begin{array}{l} S^{\phi }=\left\{\phi \in L^{2}\left(\left(0,T\right];H^{1}\left(\Omega ,\mathbb{R}\right)\right):\phi =\bar{\phi }\text{on}\partial \Omega_{\phi }\times \left(0,T\right]\right\} \end{array}$$

(7) $$\begin{array}{l} S^{r}=\left\{r\in L^{2}\left(\left(0,T\right];L^{2}\left(\Omega ,{\mathbb{R}}^{m}\right)\right)\right\} \end{array}$$

(8) $$\begin{array}{l} V^{\phi }=\left\{\nu \in H^{1}\left(\Omega ,\mathbb{R}\right):\nu =0\text{on}\partial \Omega_{\phi }\right\} \end{array}$$

(9) $$\begin{array}{l} V^{r}=\left\{\eta \in L^{2}\left(\Omega ,{\mathbb{R}}^{m}\right)\right\} \end{array}$$

Multiplying (2) and (3) by appropriate test functions, integrating over Ω and applying the divergence theorem yields the weak equations, and the statement of the weak formulation reads: ∀*t* ∈ (0, *T*], find $\left(\phi ,r\right)\in S^{\phi }\times S^{r}$ such that

(10) $$\begin{array}{ll} G^{\phi }\left[\left(\phi ,r\right),\left(\nu ,\eta \right)\right] & :=\int_{\Omega }\nu \frac{\partial \phi }{\partial t}\text{d}x+\int_{\Omega }\nabla \nu \cdot \text{D}\nabla \phi \text{d}x\\ -\int_{\Omega }\nu f\left(\phi ,r\right)\text{d}x+\int_{\partial \Omega_{q}}\nu \bar{q}\text{d}s\\ =0,\;\forall \nu \in V^{\phi } \end{array}$$

(11) $$\begin{array}{ll} G^{r}\left[\left(\phi ,r\right),\left(\nu ,\eta \right)\right] & :=\int_{\Omega }\eta \left\{\frac{\partial r}{\partial t}-g\left(\phi ,r\right)\right\}\text{d}x\\ =0,\;\forall \eta \in V^{r} \end{array}$$

### 2.2. Spatial discretization using a non-conforming finite-element scheme

A Galerkin finite-element scheme to solve the weak formulation of the monodomain problem can be stated as follows. Let $\Omega^{h}=\cup_{e=1}^{N_{\text{el}}}\Omega_{e}$ be a domain discretization where *N*_el_ is the number of elements, and all elements comply with the condition Ω_*i*_∩Ω_*j*_ = ∅ for *i* ≠ *j*. We construct finite-dimensional subspaces $S_{h}^{\phi }\subset S^{\phi }$, $S_{h}^{r}\subset S^{r}$ and $V_{h}^{\phi }\subset V^{\phi }$, $V_{h}^{r}\subset V^{r}$, to solve the following FE problem (Göktepe and Kuhl, 2009; Hurtado and Kuhl, 2014): ∀*t* ∈ (0, *T*], find $\left(\phi^{h},r^{h}\right)\in S_{h}^{\phi }\times S_{h}^{r}$ such that

$$\begin{array}{l} G^{\phi }\left[\left(\phi^{h},r^{h}\right),\left(\nu^{h},\eta^{h}\right)\right]=0,\;\forall \nu^{h}\in V_{h}^{\phi }\\ G^{r}\left[\left(\phi^{h},r^{h}\right),\left(\nu^{h},\eta^{h}\right)\right]=0,\;\forall \eta^{h}\in V^{r}. \end{array}$$

A traditional discretization FE scheme is the hexahedral isoparametric finite-element space,

$$\begin{array}{l} V_{h}^{\phi }:=\left\{\nu^{h}\in C^{0}\left(\Omega^{h},\mathbb{R}\right):\nu^{h}{|}_{\Omega_{e}}\in Q_{k}\left(\Omega_{e}\right),e=1,\ldots ,N_{\text{el}}\right\} \end{array}$$

where *Q*_*k*_(Ω_*e*_) represents the space of isoparametric functions resulting from *n*-tensor product of 1-D Lagrange polynomials of order *k*, which are defined over the standard (isoparametric) domain $\hat{\Omega }={\left[-1,1\right]}^{n}$ and mapping to a hexahedral element. We expand an element $\nu^{h}\in V_{h}^{\phi }$ as

$$\begin{array}{l} \nu^{h}\left(x\right)=\overset{N_{\text{dofs}}}{\underset{A=1}{\sum }}N_{A}\left(x\right)\nu_{A}, \end{array}$$

where {*N*_*A*_}*a* = 1, *N*_dofs_ are the basis functions, *N*_dofs_ is the number of element nodes with unknown degrees of freedom, and {ν_*A*_}*a* = 1, *N*_dofs_ are the nodal coefficients. Using the same element basis functions, we expand the trial functions as

(12) $$\begin{array}{l} \phi^{h}\left(x,t\right)=\overset{N_{\text{dofs}}}{\underset{A=1}{\sum }}N_{A}\left(x\right)u_{A}\left(t\right)+u_{\text{BC}}\left(x,t\right), \end{array}$$

where {*u*_*A*_(*t*)}*A* = 1, *N*_dofs_ correspond to the nodal values of the transmembrane potential field, and $u_{\text{BC}}\in S^{\phi }$ is a function that satisfies the boundary conditions (4), i.e., $u_{\text{BC}}=\bar{\phi }$ in ∂Ω_ϕ_ × (0, *T*]. For simplicity, and without loss of generality, in the following we assume that *u*_BC_ = 0. To construct the elements of $V_{h}^{r}$, we write

(13) $$\begin{array}{l} \eta^{h}\left(x\right)=\overset{N_{\text{el}}}{\underset{e=1}{\sum }}\overset{N_{\text{q}}}{\underset{q=1}{\sum }}M_{q}^{e}\left(x\right)\eta_{q}^{e}, \end{array}$$

where $M_{q}^{e}$ is a characteristic function defined by

(14) $$\begin{array}{l} M_{q}^{e}\left(x\right)=\begin{array}{c} 1,x\in \Omega_{e,q}\;\\ 0,x\notin \Omega_{e,q}\; \end{array} \end{array}$$

and Ω_*e,q*_ ⊂ Ω_*e*_ is the subdomain containing the *q*−quadrature point $x_{q}$, and is such that ${⋃}_{q=1}^{Nq}\Omega_{e,q}=\Omega_{e}$ and $\Omega_{e,q}\cap \Omega_{e,q^{\prime }}=\emptyset$ whenever *q* ≠ *q*′. Analogously, we expand an element $r^{h}\in S_{h}^{r}$ as

(15) $$\begin{array}{l} r^{h}\left(x,t\right)=\overset{N_{\text{el}}}{\underset{e=1}{\sum }}\overset{N_{\text{q}}}{\underset{q=1}{\sum }}M_{q}^{e}\left(x\right)r_{q}^{e}\left(t\right), \end{array}$$

where $r_{q}^{e}:\left(0,T\right]\to {\mathbb{R}}^{\text{m}}$ represents the time evolution of the state variables at the *q*-quadrature point.

In this work, we consider a non-conforming spatial-discretization scheme for the monodomain equations (Hurtado and Rojas, 2018). To this end, we rewrite the residuals as

(16) $$\begin{array}{l} G^{\phi }[(\phi ,r),(\nu ,\eta )]=\sum_{e=1}^{N_{\text{el}}}\{\int_{\Omega_{e}}\nu \frac{\partial \phi }{\partial t}\text{ d}x+\int_{\Omega_{e}}\nabla \nu \cdot \text{D}\nabla \phi \text{ d}x\\ \;\;-\int_{\Omega_{e}}\nu f(\phi ,r)\text{ d}x+\int_{\partial \Omega_{e,q}}\nu \bar{q}\text{ d}s\}, \end{array}$$

(17) $$\begin{array}{l} G^{r}\left[\left(\phi ,r\right),\left(\nu ,\eta \right)\right]=\overset{N_{\text{el}}}{\underset{e=1}{\sum }}\left\{\int_{\Omega_{e}}\eta \left\{\frac{\partial r}{\partial t}-g\left(\phi ,r\right)\right\}\text{d}x\right\}, \end{array}$$

and note that in such form, integrability of the trial and test functions and their weak derivatives is required only at the element level. We enhance the polynomial basis of $V_{h}^{\phi }$ at the element level by adding polynomial terms not included in *Q*_*k*_(Ω_*e*_). To this end, we consider the non-conforming space

$$\begin{array}{l} E_{h}^{\phi }:=\left\{\beta^{h}:\beta^{h}{|}_{\Omega_{e}}\in P_{k+m}\left(\Omega_{e}\right)\backslash Q_{k}\left(\Omega_{e}\right)\right\} \end{array}$$

where *m* ∈ ℤ_+_ and *P*_*k*+*m*_(Ω_*e*_) is the space of polynomial functions of degree *k*+*m* defined on the standard domain $\hat{\Omega }$. We then consider enhanced test functions ν^*h*^ which we expand as

(18) $$\begin{array}{l} \nu^{h}\left(x\right)=\overset{N_{\text{dofs}}}{\underset{A=1}{\sum }}N_{A}\left(x\right)\nu_{A}+\overset{N_{\text{el}}}{\underset{e=1}{\sum }}\overset{N_{\text{nc}}}{\underset{c=1}{\sum }}W_{c}^{e}\left(x\right)\beta_{c}^{e} \end{array}$$

where $\beta_{c}^{e}\in \mathbb{R}$ are coefficients, $W_{c}^{e}$ are non-conforming element basis functions, and it holds that $W_{c}^{e}=0,x\notin \Omega^{e}$. Analogously, we enhance $S_{h}^{\phi }$ with the time-dependent non-conforming space $F_{h}^{\phi }$, and expand the enhanced trial functions as

(19) $$\begin{array}{l} \phi^{h}\left(x,t\right)=u^{h}\left(x,t\right)+\alpha^{h}\left(x,t\right) \end{array}$$

where

(20) $$\begin{array}{ll} u^{h}\left(x,t\right) & :=\overset{N_{\text{dofs}}}{\underset{B=1}{\sum }}N_{B}\left(x\right)u_{B}\left(t\right) \end{array}$$

(21) $$\begin{array}{ll} \alpha^{h}\left(x,t\right) & :=\overset{N_{\text{el}}}{\underset{e=1}{\sum }}\overset{N_{\text{nc}}}{\underset{d=1}{\sum }}W_{d}^{e}\left(x\right)\alpha_{d}^{e}\left(t\right). \end{array}$$

and $\alpha_{d}^{e}:\left(0,T\right]\to \mathbb{R}$ is a time-dependent coefficient that scales the non-conforming basis functions $W_{d}^{e}$. Substitution of approximations Equations (13 15, 18, and 19) into the residuals Equations (16) and (17) yields the following semi-discrete problem: ∀*t* ∈ (0, *T*], find $\left(u^{h},\alpha^{h},r^{h}\right)\in S_{h}^{\phi }\times F_{h}^{\phi }\times S_{h}^{r}$ such that

(22) $$\begin{array}{l} \int_{\Omega }N_{A}\{{\dot{u}}^{h}+{\dot{\alpha }}^{h}\}\text{d}x+\int_{\Omega }\nabla N_{A}\cdot \text{D}\nabla \{u^{h}+\alpha^{h}\}\text{d}x-\int_{\Omega }N_{A}f(u^{h}\\ \;+\alpha^{h},r^{h})\text{d}x+\int_{\partial \Omega_{q}}N_{A}\bar{q}\text{ d}s=0,\;A=1,\ldots ,N_{\text{dofs}}, \end{array}$$

(23) $$\begin{array}{l} \int_{\Omega^{e}}W_{c}^{e}\left\{{\dot{u}}^{h}+{\dot{\alpha }}^{h}\right\}\text{d}x+\int_{\Omega^{e}}\nabla W_{c}^{e}\cdot \text{D}\nabla \left\{u^{h}+\alpha^{h}\right\}\text{d}x\\ \;-\int_{\Omega^{e}}W_{c}^{e}f\left(u^{h}+\alpha^{h},r^{h}\right)\text{d}x=0,\;e=1,\ldots ,N_{\text{el}};c=1,\ldots ,N_{\text{nc}}, \end{array}$$

(24) $$\begin{array}{l} \int_{\Omega^{e}}M_{q}^{e}\left\{{\dot{r}}^{h}-g\left(u^{h}+\alpha^{h},r^{h}\right)\right\}\text{d}x=0,\;e=1,\ldots ,N_{\text{el}};q=1,\ldots ,N_{\text{q}} \end{array}$$

### 2.3. Semi-implicit temporal discretization

To integrate (22), (23) and (24) in time, we consider partitioning the time interval into [0, …, *t*_*n*_, *t*_*n*+1_, …, *T*], and approximate the time-dependent coefficients □(*t*_*n*_) ≈ □_*n*_. For a generic time interval [*t*_*n*_, *t*_*n*+1_] we define Δ*t*: = *t*_*n*+1_−*t*_*n*_. We further group the expansion coefficients into vectors, and write

(25) $$\begin{array}{l} \begin{array}{c} u_{n}={\left[u_{n,1},\ldots ,u_{n,N_{\text{dofs}}}\right]}^{T},\;\alpha_{n}^{e}={\left[\alpha_{n,1}^{e},\ldots ,\alpha_{n,N_{\text{nc}}}^{e}\right]}^{T},\\ r_{n}^{e}={\left[r_{n,1}^{e},\ldots ,r_{n,N_{\text{q}}}^{e}\right]}^{T} \end{array} \end{array}$$

Following a semi-implicit (SI) time-integration approach (Whiteley, 2006), time derivatives are replaced by the finite-difference approximation

(26) $$\begin{array}{l} \dot{□}\left(t_{n+1}\right)\approx \frac{{□}_{n+1}-{□}_{n}}{\Delta t}. \end{array}$$

Diffusive terms in Equations (22) and (23) are evaluated at *t* = *t*_*n*+1_ and the reaction terms are evaluated at *t* = *t*_*n*_. Evolution Equation (24) were integrated using an explicit Forward-Euler scheme. As a result, the incremental time update for *t* = *t*_*n*+1_ reads: Given $u_{n},{\left\{\alpha_{n}^{e},r_{n}^{e}\right\}}_{e=1,\ldots ,N_{\text{el}}}$, find $u_{n+1},{\left\{\alpha_{n+1}^{e},r_{n+1}^{e}\right\}}_{e=1,\ldots ,N_{\text{el}}}$ such that

(27) $$\begin{array}{l} \overset{N_{\text{dofs}}}{\underset{B=1}{\sum }}\left\{\int_{\Omega }\frac{N_{A}N_{B}}{\Delta t}+\int_{\Omega }\nabla N_{A}\cdot \text{D}\nabla N_{B}\right\}u_{n+1,B}\\ \;+\overset{N_{\text{el}}}{\underset{e=1}{\sum }}\overset{N_{\text{nc}}}{\underset{d=1}{\sum }}\left\{\int_{\Omega }\frac{N_{A}W_{d}^{e}}{\Delta t}+\int_{\Omega }\nabla N_{A}\cdot \text{D}\nabla W_{d}^{e}\right\}\alpha_{n+1,d}^{e}\\ \;-\left\{\int_{\Omega }\frac{N_{A}}{\Delta t}\left\{u_{n}^{h}+\alpha_{n}^{h}\right\}+\int_{\Omega }N_{A}f\left(u_{n}^{h}+\alpha_{n}^{h},r_{n}^{h}\right)\right\}=0,\\ \;A=1,\ldots ,N_{\text{dofs}}, \end{array}$$

(28) $$\begin{array}{l} \overset{b=1}{\underset{N_{en}}{\sum^{\text{​}}}}\underset{=:L_{cb}^{e}}{\underset{︸}{\left\{{\int^{\text{​}}}_{\Omega^{e}}\frac{W_{c}^{e}N_{b}^{e}}{\Delta t}+{\int^{\text{​}}}_{\Omega^{e}}\nabla W_{c}^{e}\cdot \text{D}\nabla N_{b}^{e}\right\}}}u_{n+1,b}^{e}\\ \;+\overset{d=1}{\underset{N_{nc}}{\sum^{\text{​}}}}\underset{{}_{=:K_{\alpha_{cd}}^{e}}}{\underset{︸}{\left\{{\int^{\text{​}}}_{\Omega^{e}}\frac{W_{c}^{e}W_{d}^{e}}{\Delta t}+{\int^{\text{​}}}_{\Omega^{e}}\nabla W_{c}^{e}\cdot \text{D}\nabla W_{d}^{e}\right\}}}\alpha_{n+1,d}^{e}\\ \;-\underset{=:p_{\alpha_{c}}^{e}}{\underset{︸}{\left\{{\int^{\text{​}}}_{\Omega^{e}}\frac{W_{c}^{e}}{\Delta t}\{u_{n}^{h}+\alpha_{n}^{h}\}+{\int^{\text{​}}}_{\Omega^{e}}W_{c}^{e}f(u_{n}^{h}+\alpha_{n}^{h},r_{n}^{h})\right\}}}=0\\ \;e=1,\ldots ,N_{el};c=1,\ldots ,N_{nc} \end{array}$$

(29) $$\begin{array}{l} \int_{\Omega^{e}}M_{q}^{e}\left\{\overset{N_{q}}{\underset{s=1}{\sum }}M_{s}^{e}\frac{r_{n+1,s}^{e}-r_{n,s}^{e}}{\Delta t}-g\left(u_{n}^{h}+\alpha_{n}^{h},r_{n}^{h}\right)\right\}\text{d}x=0,\\ \;e=1,\ldots ,N_{\text{el}};q=1,\ldots ,N_{q}, \end{array}$$

where $N_{b}^{e}:={N_{B}|}_{\Omega^{e}}$ is the restriction of the basis function to the local element domain, and $u_{b}^{e}$ is the corresponding nodal value, where lowercase letters indicate the local degree of freedom *b* corresponding to its global counterpart *B*. At this point, we note that Equation (28) can be written in matrix form as

$$\begin{array}{l} L^{e}u_{n+1}^{e}+K_{\alpha }^{e}\alpha_{n+1}^{e}-p^{e}\left(u_{n}^{e},\alpha_{n}^{e},r_{n}^{e}\right)=0, \end{array}$$

for *e* = 1, …, *N*_el_, from where we define the time update for the element non-conforming coefficient vector as

(30) $$\begin{array}{ll} \alpha_{n+1}^{e,*}\left(u_{n+1}^{e};u_{n}^{e},\alpha_{n}^{e},r_{n}^{e}\right) & :={\left\{K_{\alpha }^{e}\right\}}^{-1}p_{\alpha }^{e}\left(u_{n}^{e},\alpha_{n}^{e},r_{n}^{e}\right)\\ -{\left\{K_{\alpha }^{e}\right\}}^{-1}L^{e}u_{n+1}^{e} \end{array}$$

which is computed exclusively using element-level variables, given the element vector $u_{n+1}^{e}$. To update the gating-variable field, we note from Equation (14) that Equation (29) can be solved point-wise at each quadrature point $x_{q}$ inside an element, and thus is equivalent to writing

$$\begin{array}{l} \frac{r_{q,n+1}^{e}-r_{q,n}^{e}}{\Delta t}-g\left(u_{n}^{h}\left(x_{q}\right)+\alpha_{n}^{h}\left(x_{q}\right),r_{q,n}^{e}\right)=0,\\ \;e=1,\ldots ,N_{\text{el}};q=1,\ldots ,N_{q}, \end{array}$$

from which the (explicit) time update for the gating variables can be solved at the quadrature-point level as

(31) $$\begin{array}{l} r_{q,n+1}^{e,*}\left(u_{n}^{e},\alpha_{n}^{e},r_{n}^{e}\right):=r_{q,n}^{e}+\Delta tg\left(u_{n}^{h}\left(x_{q}\right)+\alpha_{n}^{h}\left(x_{q}\right),r_{q,n}^{e}\right). \end{array}$$

We now turn to residual Equation (27), and note that it can be constructed by assembling element-level nodal contributions defined by

(32) $$\begin{array}{ll} R_{a}^{u,e} & :=\overset{N_{\text{en}}}{\underset{b=1}{\sum }}{\underset{︸}{\left\{\int_{\Omega^{e}}\frac{N_{a}N_{b}}{\Delta t}+\int_{\Omega^{e}}\nabla N_{a}\cdot \text{D}\nabla N_{b}\right\}}}_{:=K_{u_{ab}}^{e}}u_{n+1,b}^{e}\\ \;+\overset{N_{\text{en}}}{\underset{b=1}{\sum }}{\underset{︸}{\left\{\int_{\Omega^{e}}\frac{N_{a}W_{d}}{\Delta t}+\int_{\Omega^{e}}\nabla N_{a}\cdot \text{D}\nabla W_{d}\right\}}}_{L_{ad}^{eT}}\alpha_{n+1,d}^{e}\\ \;-{\underset{︸}{\left\{\int_{\Omega^{e}}\frac{N_{a}}{\Delta t}\left\{u_{n}^{h}+\alpha_{n}^{h}\right\}+\int_{\Omega^{e}}N_{a}f\left(u_{n}^{h}+\alpha_{n}^{h},r_{n}^{h}\right)\right\}}}_{:=p_{u_{a}}^{e}}, \end{array}$$

which can also be written in matrix form at the element level as

(33) $$\begin{array}{l} R^{u,e}=K_{u}^{e}u_{n+1}^{e}+L^{eT}\alpha_{n+1}^{e}-p_{u}^{e}\left(u_{n}^{e},\alpha_{n}^{e},r_{n}^{e}\right). \end{array}$$

Substituting update Equation (30) into Equation (33), we obtain an element residual that only depends on $u_{n+1}^{e}$ that reads

(34) $$\begin{array}{l} R^{u,e}={\underset{︸}{\left(K_{u}^{e}-L^{eT}{\left\{K_{\alpha }^{e}\right\}}^{-1}L^{e}\right)}}_{A^{e}}u_{n+1}^{e}\\ \;+{\underset{︸}{L^{eT}{\left\{K_{\alpha }^{e}\right\}}^{-1}p_{\alpha }^{e}\left(u_{n}^{e},\alpha_{n}^{e},r_{n}^{e}\right)-p_{u}^{e}\left(u_{n}^{e},\alpha_{n}^{e},r_{n}^{e}\right)}}_{b_{n}^{e}\left(u_{n}^{e},\alpha_{n}^{e},r_{n}^{e}\right)} \end{array}$$

As a consequence, solving residual Equation (27) is equivalent to solving the matrix linear system

(35) $$\begin{array}{l} Au_{n+1}+b_{n}=0 \end{array}$$

where ***A*** and $b_{n}$ are the global matrix and vector assembled from element contributions defined in Equation (34). We note that Equation (35) defines the time update for the global potential vector

(36) $$\begin{array}{l} u_{n+1}^{*}\left(u_{n},{\left\{\alpha_{n}^{e},r_{n}^{e}\right\}}_{e=1,\ldots ,N_{\text{el}}}\right):=-A^{-1}b_{n} \end{array}$$

We remark that matrix ***A*** does not depend on the coefficient vectors, and therefore will take the same values for all time steps. Thus, it can be computed on a initialization stage, inverted and stored for later use in updating the potential vector. For the sake of clarity, the steps for the solving the semi-implicit scheme are summarized in Algorithm 1.

**Algorithm 1:**
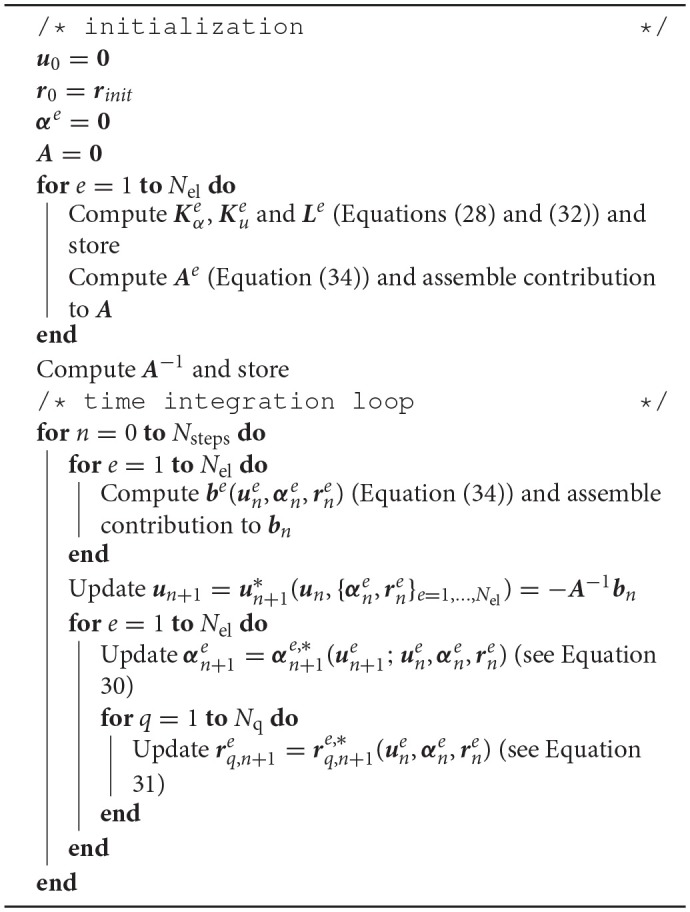
Solution algorithm

### 2.4. The Q1NC element

We materialize the non-conforming scheme defined in the previous section using incompatible-modes basis functions (Wilson et al., 1973; Taylor et al., 1976), which enhance Q1 elements. We recall that the isoparametric basis functions for Q1 3D (solid) elements are

$$\begin{array}{l} {\hat{N}}_{1}=\frac{1}{8}\left(1-\xi_{1}\right)\left(1-\xi_{2}\right)\left(1-\xi_{3}\right),\;{\hat{N}}_{2}=\frac{1}{8}\left(1+\xi_{1}\right)\left(1-\xi_{2}\right)\left(1-\xi_{3}\right),\\ {\hat{N}}_{3}=\frac{1}{8}\left(1+\xi_{1}\right)\left(1+\xi_{2}\right)\left(1-\xi_{3}\right),\;{\hat{N}}_{4}=\frac{1}{8}\left(1-\xi_{1}\right)\left(1+\xi_{2}\right)\left(1-\xi_{3}\right),\\ {\hat{N}}_{5}=\frac{1}{8}\left(1-\xi_{1}\right)\left(1-\xi_{2}\right)\left(1+\xi_{3}\right),\;{\hat{N}}_{6}=\frac{1}{8}\left(1+\xi_{1}\right)\left(1-\xi_{2}\right)\left(1+\xi_{3}\right),\\ {\hat{N}}_{7}=\frac{1}{8}\left(1+\xi_{1}\right)\left(1+\xi_{2}\right)\left(1+\xi_{3}\right),\;{\hat{N}}_{8}=\frac{1}{8}\left(1-\xi_{1}\right)\left(1+\xi_{2}\right)\left(1+\xi_{3}\right), \end{array}$$

where $\left(\xi_{1},\xi_{2},\xi_{3}\right)\in \hat{\Omega }:={\left[-1,1\right]}^{3}$, and

$$\begin{array}{l} N_{a}^{e}={\hat{N}}_{a}˙{\hat{x}}^{-1} \end{array}$$

with

$$\begin{array}{l} \hat{x}=\overset{8}{\underset{a=1}{\sum }}{\hat{N}}_{a}x_{a}^{e}, \end{array}$$

where $x_{a}^{e}$ is the vector with nodal coordinates. Incompatible modes enhance the Q1(Ω^*e*^) element basis by adding basis functions ${\left\{M_{c}^{e}\right\}}_{c=1,2,3}$, with $M_{c}^{e}={\hat{M}}_{c}˙{\hat{x}}^{-1}$, where

$$\begin{array}{l} {\hat{M}}_{1}=1-{\left(\xi_{1}\right)}^{2},\;{\hat{M}}_{2}=1-{\left(\xi_{2}\right)}^{2},\;{\hat{M}}_{3}=1-{\left(\xi_{3}\right)}^{2} \end{array}$$

for $\left(\xi_{1},\xi_{2},\xi_{3}\right)\in \hat{\Omega }$. Table 1 details the number of DOFs used for the 3D elements considered in this work. Integrals have been approximated using Gaussian quadrature on the standard domain. Table 1 reports the quadrature rules employed in the numerical integration of Q1, Q1NC and Q2 element implementations.

**Table 1:** Element DOFs and quadrature rules employed in numerical integration of residuals and tangents.

	**Element DOFs**	**Quadrature rule**
Q1	8 DOFs	2 × 2 × 2 = 8-point
Q1NC	8 DOFs + 3 IMs	2 × 2 × 2 = 8-point
Q2	27 DOFs	3 × 3 × 3 = 27-point

*DOFs, degrees of freedom; NC, incompatible mode (internal variable)*.

### 2.5. The modified Aliev-Panfilov model for transmembrane ionic current

All simulations considered the modified Aliev-Panfilov model, which accounts for physiological voltage upstroke slopes and conduction velocities (Aliev and Panfilov, 1996; Hurtado et al., 2016), whose expressions are described below for completeness:

(37) $$\begin{array}{l} f\left(\phi ,r\right)=c_{1}\phi \left(\phi -\alpha \right)\left(1-\phi \right)-c_{2}r\phi \end{array}$$

(38) $$\begin{array}{l} g\left(\phi ,r\right)=\left(\gamma +\frac{\mu_{1}r}{\mu_{2}+\phi }\right)\left(-r-c_{2}\phi \left(\phi -b-1\right)\right) \end{array}$$

where *c*_1_, *c*_2_, α, γ, μ_1_, μ_2_ and *b* are constants, whose values are included in Table 2, and are the same employed by Hurtado and Rojas (2018). To account for a steady-state regime, initial values of the recovery value where set to *r* = 0.1146.

**Table 2:** Parameter values for the modified Aliev-Panfilov model.

**α**	***c*_1_**	***c*_2_**	***μ*_1_**	***μ*_2_**	***b***	**γ**	***V*_*r*_[mV]**	***V*_*p*_[mV]**
0.05	52	8	0.1	0.3	0.25	0.002	−85	15

## 3. Results

Finite-element simulations using Q1, Q2, and Q1NC element formulations were implemented for the FI and SI time-integration schemes described in the previous section in an enhanced version of FEAP (Taylor, 2014).

### 3.1. Plane-wave tests on CV and CT

**Figure 1:**
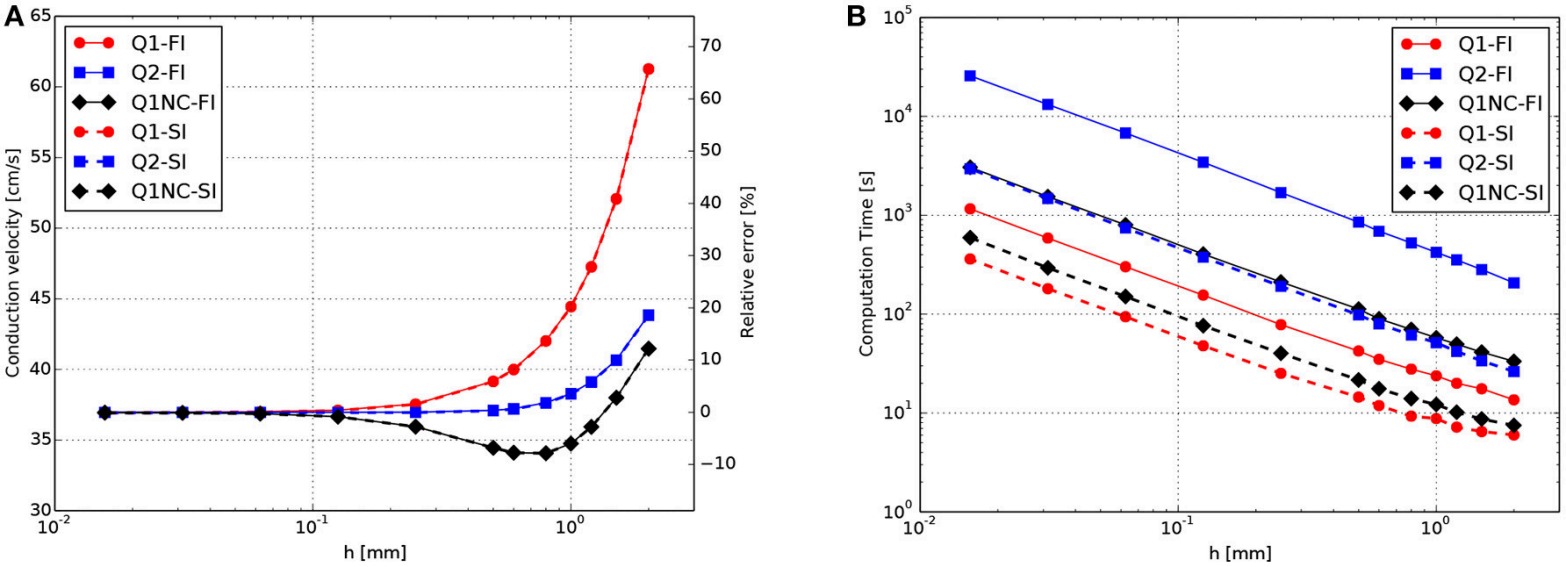
CV tests for plane-waves propagating on a 3D bar for FI and SI schemes on different element formulations. **(A)** Convergence study of CV as a function of the mesh size *h*. **(B)** Computational effort in terms of CT as a function of *h*.

A 3D cardiac rod with a total length of 25 mm was discretized using regular hexahedral elements with a uniform element size, with the exception of elements adjacent to the boundary where the size was at times smaller to fit the geometry. To study the effect of the element size, simulations were carried out with mesh sizes ranging from *h* = 2mm to *h* = 0.0156mm. A zero-flux boundary condition was assumed for all boundary surfaces, with exception of the left end of the rod which was stimulated with a normalized external current of 20mV/ms, which corresponds to 28, 000μA/cm^3^, for 2ms to elicit a plane traveling wave along the direction of the rod. A time-step size of 0.001ms was set for all simulations, which is small when compared to standard cardiac simulations using the selected ionic model (Hurtado et al., 2016). Such small time-step size is chosen to minimize the contribution of the temporal discretization error to the overall numerical error. To compute the CV, we tracked the voltage evolution on *x*_1_ = 18mm and *x*_2_ = 22mm and recorded the activation time, which is defined as the time when the ϕ>0.5 for the first time at a certain point. Then, the CV was calculated as the difference between *x*_2_ and *x*_1_ divided by the difference in the activation time. The results for the CV for different element sizes are shown in Figure 1A. All formulations converged to a CV = 36.9cm/*s* as the mesh size approached *h* = 0.0156mm. CV monotonically decreased as mesh size was decreased for Q1 and Q2 formulations. The computational effort of simulations in terms of CT is reported in Figure 1B. We observe that the computational demand of simulations monotonically increases as the mesh size decreases, independently of the element formulation. We do observe, however, that the FI time-integration scheme always results in higher CT than the SI scheme for all element formulations considered.

**Figure 2:**
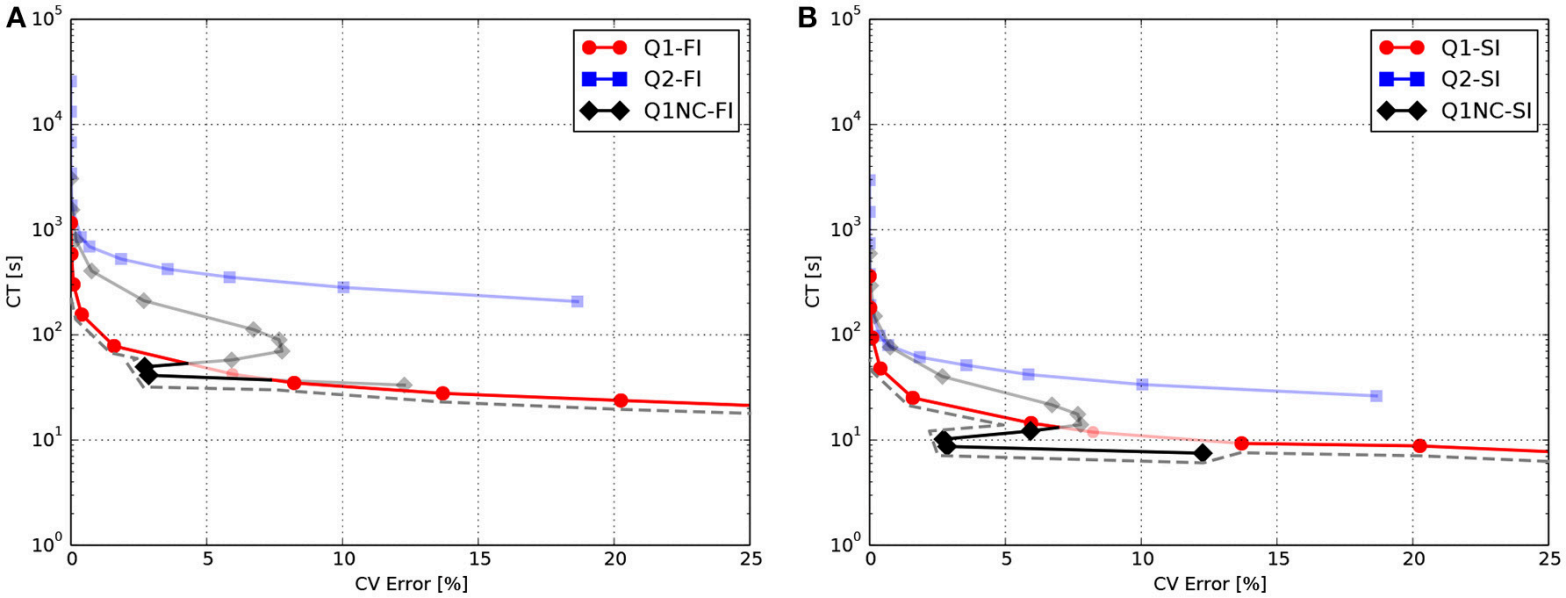
Accuracy-efficiency analysis: Computation time vs. conduction-velocity error for the different spatial discretization schemes using **(A)** fully-implicit time integration, and **(B)** semi-implicit time integration. Dashed gray line displays the Pareto frontier, which encompasses optimal cases. Suboptimal combinations are shown in transparent color.

To facilitate the analysis of the accuracy-efficiency trade-off of the different schemes studied, Figure 2 shows the CT vs. the error in CV for the Q1, Q2, and Q1NC formulations for both the implicit and semi-implicit time updates. Since we seek to minimize two objective functions, namely the CT and the CV error, the Pareto frontier, defined as the set of choices that are Pareto-efficient, is included in each subfigure. The subset of the Pareto-efficient cases that correspond to the Q1NC formulation are {1.2, 1.5}[mm] and {1.0, 1.2, 1.5, 2.0}[mm] for the FI and SI cases, respectively.

### 3.2. Benchmark simulations on a cardiac cuboid

**Figure 3:**
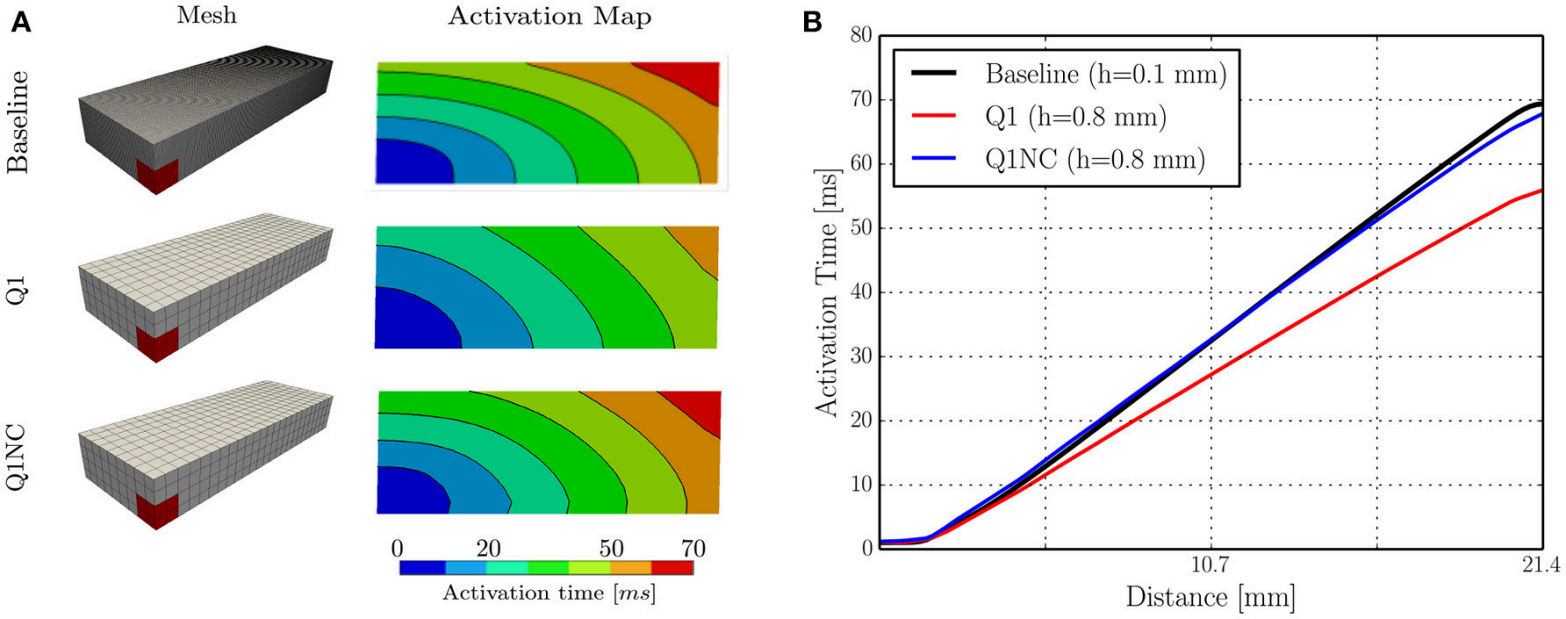
Numerical simulations on cuboid benchmark test **(A)** Meshes and activation maps, and **(B)** Activation time profile along the cuboid diagonal.

We studied the behavior of the SINCFES using as a second test case the benchmark study on a cardiac cuboid developed by Niederer et al. (2011b), and adapted to the case of the Aliev-Panfilov model by Hurtado et al. (2016). To this end, we consider a cuboid domain with dimensions of 20 × 7 × 3mm with cardiac fibers oriented in the longest axis direction. A subdomain with dimensions 1.5 × 1.5 × 1.5mm located at one of the corners of the cuboid was stimulated with an electrical current density of 50, 000/cm^3^ for 2ms. The normalized longitudinal and transversal conductivities were 0.0952 and 0.0126mm^2^/ms, respectively. Figure 3A shows the activation map and isochrones obtained on a plane that contains opposite corners in the diagonal, as defined in Niederer et al. (2011b), for a fine (Baseline) and coarse discretization using Q1 elements, and for the same coarse discretization using Q1NC elements. We note that the Q1NC case with mesh size *h* = 0.8mm resulted in an activation map and isochrones similar to the baseline case, defined as a Q1 model with mesh size *h* = 0.1mm. In contrast, the activation map delivered by the Q1 coarse-mesh case with mesh size *h* = 0.8mm largely differed from the baseline case, delivering a less curved wave-front profile. Figure 3 displays the activation time values along the diagonal of the cuboid for the three cases under study. We observe that the Q1NC case closely follows the baseline case, whereas the Q1 coarse-mesh case resulted in shorter activation times at all locations along the diagonal. As a reference, the CT for the Baseline (Q1 fine), Q1NC and Q1 cases were 122, 341, 344, and 184 s, respectively, which is equivalent to a CT ratio of 665 : 2 : 1.

### 3.3. Biventricular human heart simulations

**Figure 4:**
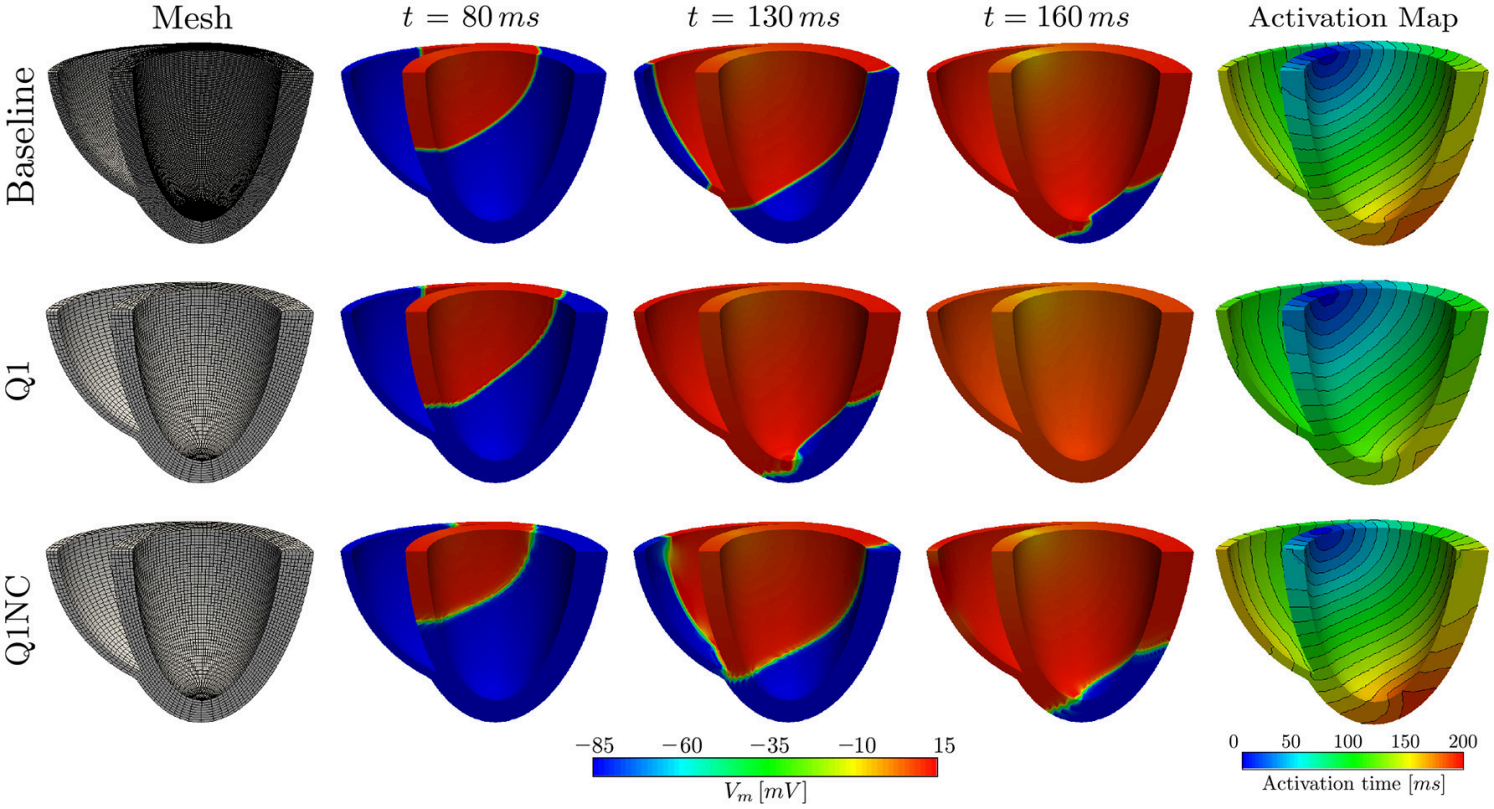
Numerical simulations on human biventricular idealized geometries. The Q1NC model displays a propagating wave similar to the baseline case during the ventricular activation sequence, whereas the Q1 model hastens the electrical stimulation ahead of the baseline case.

To study the potential of the Q1NC-SI formulation in whole-heart cardiac simulations, we modeled the propagation of an action potential on an idealized human biventricular domain stimulated at the atrio-ventricular node. The heart biventricular geometry was generated from two truncated ellipsoids (Streeter and Hanna, 1973), and later discretized using non-regular hexahedral elements. For the baseline case, a size-varying mesh with average characteristic length of 0.48mm was employed. A coarse mesh with average element length of 1.0mm was also considered for two additional cases with Q1 and Q1NC element formulations, see left column of Figure 4 for a representation of the biventricular meshes. All three cases considered the same initial boundary conditions and time step size of 0.001ms. The transmembrane potential distribution at different time instants during ventricular activation is depicted in Figure 4. We clearly observe that, as time elapses, the action-potential wave front of the Q1NC case is very similar to the Baseline case, whereas the Q1 case results in a wave front that propagates faster than the other two models due to the artificially high CV. The last column in Figure 4 shows the activation maps, where we observe that isochrones for the Baseline and Q1NC cases are very similar, and they both differ from the Q1 case. Biventricular simulations were ran in a HPC cluster with 128 GB of RAM memory using 32 processors using the parallel implementation of the code FEAP (Taylor, 2014). The CT for the baseline, the Q1NC and the Q1 simulation were 1805, 452 and 154 min respectively, which is equivalent to a CT ratio of 18 : 3 : 1.

### 3.4. Spiral wave simulations

**Figure 5:**
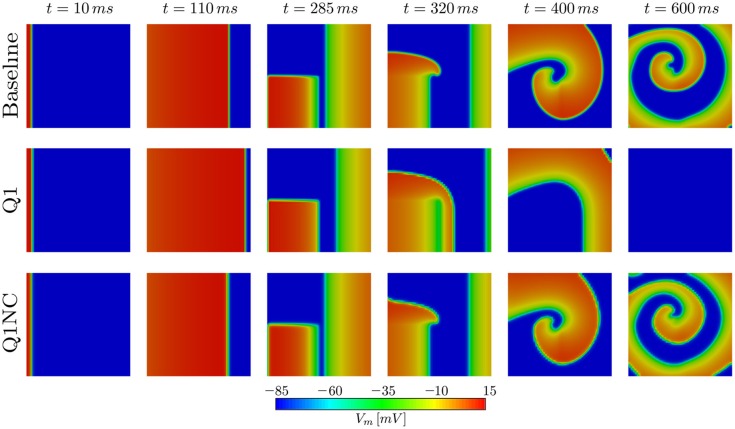
Spiral generation simulation in a 2D slab. Due to the higher CV, the Q1 case (coarse mesh) cannot capture the genesis of the spiral wave.

To assess the performance of the proposed non-conforming scheme in the simulation of spiral waves, we considered a 50 × 50mm cardiac domain excited by means of an S1–S2 stimulation protocol. To this end, we first applied a surface stimulus (S1) of 12mV/(msmm^2^) for 2ms on the border defined by *x* = 0 to create a plane wave. After 280ms, we applied a second stimulus (S2) of 15mV/(msmm^3^) in the quadrant *x* < 25, *y* < 25mm for 5ms, which resulted in the formation of a spiral wave (Costabal et al., 2017). This S1–S2 model was solved using three numerical models: a fine mesh with element size *h* = 0.1mm using Q1 elements (Baseline), a coarse mesh with element size *h* = 1mm using Q1 elements (Q1), and a coarse mesh with *h* = 1mm using the proposed non-conforming element formulation (Q1NC). In all cases, we considered a semi-implicit time update with time-step size Δ*t* = 0.005ms. Figure 5 shows the distribution of the transmembrane potential of the three models under study for several time instants. We note that at early times (*t* = 110ms) the Q1 case displays a wave front that advanced considerably faster than the baseline and Q1NC cases. At *t* = 400ms a spiral wave formed in the Baseline and Q1NC cases, whereas for the Q1 case a curved wave front propagated in the outward direction but did not create a spiral. At a later instant (*t* = 600ms), a spiral was steadily rotating in the Baseline and Q1NC cases, constantly reexciting tissue, whereas in the Q1 case cardiac tissue was found under complete rest, and no electrical activity was observed.

## 4. Discussion

In this work we have studied the features and advantages of a novel SINCFES in the solution of the monodomain model of cardiac electrophysiology. From plane-wave CV tests we note that the FI and SI schemes yield similar results for the conduction velocity for the time-step size employed, see Figure 1A. This is expected, as the time-step size considered here is small compared to standard values employed in numerical simulations (Krishnamoorthi et al., 2013). Interestingly, we observe that in the case of mesh sizes *h* < 0.6*mm*, the Q1, Q2, and Q1NC element formulations delivered very similar results in terms of CV error. For the cases where *h*>0.6*mm*, the CV error incurred by the Q1 formulation grows at a much faster rate than the Q2 and Q1NC formulations. An interesting result that deserves further study is the convergence trend of the Q1NC formulation, as it is not monotonically convergent in the whole range of mesh sizes studied, and it reverts the sign of the CV error in a bounded interval of mesh sizes. A similar convergence trend has been reported in the literature for standard FE discretizations, in the context of mass-lumping techniques (Pezzuto et al., 2016), which suggest as future work a more detailed study of the effect of NC spatial discretization schemes on the stiffness and mass matrices that govern the dynamics of the problem. To better analyze the accuracy-efficiency trade-off for each scheme, we constructed CT vs CV-error plots, where the Pareto frontier has been identified. We conclude that the SINCFES delivers Pareto-optimal results for cases with mesh size in the range of {1.0, 1.2, 1.5, 2.0}[mm]. For smaller mesh sizes, traditional Q1 formulations deliver better combinations of CT and CV-error than Q1NC and Q2. It is interesting to note that, in general, Q2 elements are less efficient than the Q1 and Q1NC elements from a Pareto-optimality viewpoint for the whole range of mesh sizes studied. We also note that these conclusions are particular to a plane-wave propagation case, where anisotropy of conductivity and curvature of propagating wavefronts are absent.

We further studied the performance of Q1NC elements using a benchmark problem on a cuboid cardiac domain (Niederer et al., 2011b). Our simulations showed that the Q1NC formulation on a coarse mesh (*h* = 0.8mm) can result in activation maps that are similar to those obtained on fine meshes using Q1 (*h* = 0.1mm), adequately capturing the anisotropic conduction of the propagating waves, see Figure 3A. An analysis of the activation-time profile along the cuboid diagonal shows that the Q1NC scheme delivers an accurate conduction velocity, which is comparable to Q1 meshes with mesh sizes that are 8 times smaller, see Figure 3B. This result confirms the ability of Q1NC elements to capture the propagation of electrical waves in anisotropic media with good accuracy at significantly reduced CTs. In contrast, Q1 coarse-mesh simulations resulted in markedly higher conduction velocity profiles, and did poorly in capturing the anisotropic propagation of wavefronts when compared to the Q1NC formulation.

Numerical simulations on a biventricular domain showed that our non-conforming scheme can be effectively used in unstructured meshes of idealized anatomical geometries of the heart, see Figure 4. Similarly to the cardiac rod case, a coarse mesh using Q1NC elements performs much better than a simulation using standard Q1 elements on the same discretization level, as it predicts more accurately the wavefront propagation pattern, when compared to the baseline case. This conclusion is also reached from observing the resulting activation maps, where the spatial distribution and curved shape of isochrones in the Q1NC and baseline are similar, whereas the Q1 formulation delivers an isochrone distribution with lower activation-time values. We note here that this study considered an idealized and smooth geometrical representation of the ventricles of the human heart, useful for numerical verification purposes. It is important to note that such idealized domain does not include important anatomical structures such as the intricate endocardial surface, papillary muscles, and Purkinje network, that are currently included in advanced heart models (Ponnaluri et al., 2016; Sahli Costabal et al., 2016). Future work should focus in understanding how non-conforming formulations can handle such fine-scale anatomical details and structures.

The performance of the SINCFES was studied in the simulation of spiral waves. Remarkably, a very coarse mesh using Q1NC elements is capable to correctly produce, and sustain in time, a spiral wave, whereas a standard Q1 formulation using the same mesh size results in no activation of cardiac tissue. The ability of SINCFES to reproduce spiral wave formation and dynamics is a key result of this work, as it shows that the method is *physically* more accurate than standard FE formulations for coarse discretizations. This result can be explained by the reduced dependance of the CV on the mesh size, and highlights the potential of the SINCFES in the simulation of cardiac arrhythmias, the main clinical focus of cardiac electrophysiology simulations. While spiral patterns and dynamics obtained with the Q1NC formulation are very similar to the baseline results, a time delay is observed for the former, which resulted in differences in the spatial distribution of the transmembrane voltage, see last column of Figure 5. Such delay, which can ultimately be attributed to differences in the local CV, has also been observed in studies employing very high-order space-time formulations (Coudière and Turpault, 2017), confirming that state-of-the-art simulations of spirals using standard values of mesh size and time step are also affected by this time delay. Despite this persistent numerical error, we believe that the focus of future studies should be in recovering the overall dynamical features of spirals, i.e., spiral tip trajectories (Fenton and Karma, 1998; Gizzi et al., 2013).

We close by noting that while whole-heart simulations reported in the literature predominantly employ tetrahedral discretizations, effective methods for generating patient-specific hexahedral meshes are currently available (Lamata et al., 2011). Further, hexahedral meshes have gained great attention in the context of cardiac simulations, as the numerical performance of hexahedral elements is superior to tetrahedral elements when solving mechanics of the heart, particularly under the assumption of incompressible and quasi-incompressible regimes (Hadjicharalambous et al., 2014). As a conclusion, a natural continuation of this work is the application of non-conforming schemes in the solution of electromechanical models of the heart (Nash and Panfilov, 2004). One important reason for mesh-coarsening FE models of the heart is to reduce the number of DOFs, which in the case of electromechanical cardiac models is much larger than in pure electrophysiological simulations, as displacement, fiber stretch/stress variables, and the non-linearity of tissue constitutive models drastically increase the dimensionality and computational effort needed to solve the governing equations (Göktepe and Kuhl, 2010; Hurtado et al., 2017).

## Author contributions

JJ and DH developed the theoretical framework, numerical schemes and computer algorithms. DH designed the numerical experiments. JJ coded the implementation and ran simulations. JJ and DH wrote the manuscript draft. DH reviewed the final version of the manuscript.

### Conflict of interest statement

The authors declare that the research was conducted in the absence of any commercial or financial relationships that could be construed as a potential conflict of interest.
